# Vulnerability assessment of nearshore clam habitat subject to storm waves and surge

**DOI:** 10.1038/s41598-020-80863-4

**Published:** 2021-01-12

**Authors:** Yao Zhang, Gang Wang, Qingjie Li, Wanru Huang, Xunan Liu, Chen Chen, Xiaoyong Shi, Jinhai Zheng

**Affiliations:** 1 National Marine Hazard Mitigation Service, Ministry of Natural Resources, Beijing, 100000 China; 2 Hohai University, Nanjing, 210098 China; 3 Yantai Marine Environment Monitoring Center, Yantai, 264006 China

**Keywords:** Ecology, Ecology, Environmental sciences, Hydrology, Natural hazards, Ocean sciences, Engineering

## Abstract

Present work studied the lesion mechanism of coastal clam and its vulnerability assessment subject to the hydrodynamic disturbance of extreme storm events. A clam habitat at the northeast coast of China was chosen for the demonstration study. Relocation failure after passive transport due to excessive substrate erosion or suffocation in anoxic burial under overburdening sedimentation was identified the major cause of negative biomass responses during the storm. Based on the biological propensity and physiological sensitivity of the clam, a tunable loss probability function correlating the mortality with the shell length and the seabed change was proposed. A hydrodynamic model was then adopted to compute the sediment transport and net changes in the seafloor in response to the comprehensive process of storm waves and surge. The spatial distribution of the damage states was evaluated based on the numerical results incorporating the loss probability function. The estimated damage was mainly concentrated along the wave shoaling and breaking belts parallel to the shoreline. High surge levels pushed the “damage belt” shoreward, in which case large waves were able to propagate close to the shoreline before breaking. The scientific findings are helpful to better understand the vulnerability of the clam habitat to the storm disturbance. The study result as well provides a practical methodology of the storm risk assessment for benthic communities in broader ecological and geophysical scopes. The methodology are expected to be further validated and improved by more widespread sampling on coastal ecosystem or mariculture that will withstand future storms.

## Introduction

Storm waves and surge are most destructive marine disasters for human activities in coastal regions where a large number of cities, industries and facilities are located. China’s annual direct economic loss from marine disasters averages 1.5 billion US dollar, 90% of which is caused by the extreme hydrodynamic loads during tropical and subtropical cyclones. The rapid development of coastal areas has hitherto led to land subsidence, declining permeability, and reduced wetland vegetation, which has consistently decreased disaster adaption. Meanwhile, the intensity and frequency of global storms are expected to increase in response to the changing climate and temperature rise of the ocean upper layer^1,2^. Therefore, the disaster risk and infrastructural vulnerability to storms attract substantial research efforts. Besides the physical damage to human communities, hurricane waves and surge may bring great changes to the littoral morphology accompanied by widespread impacts to coastal infaunal assemblages^3–5^. In contrast, studies on better understanding these ecological implications are insufficient and lag behind^6–9^, although critical for the proactively managed disaster resilience.

**Figure 1:**
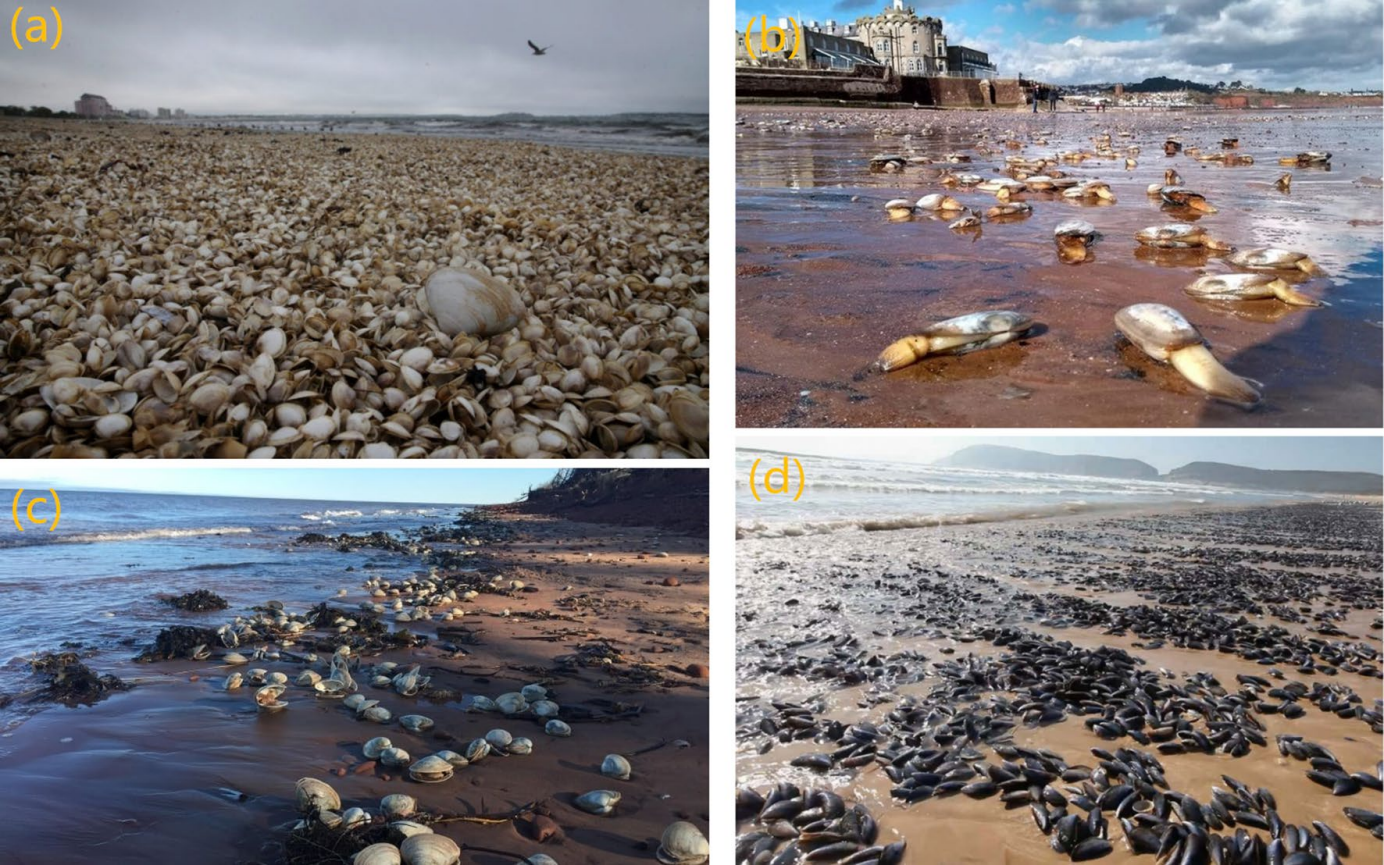
Shellfish washed up on (**a**) Revere Beach, United States in July 2018, *© The Boston Globe*; (**b**) Paignton Beach, UK during the storm Emma in March 2018, *© DevonLive*; (**c**) the beach at Robinsons Island Canada during the late November storm surge 2018, *© CBC News*; (**d**) Rodderg Beach, South Africa in December 2014, *© Earth Touch News*.

Benthos and epibenthos are highly susceptible to natural or anthropogenic disturbance. Terrestrial input, hydrodynamics, offshore dredging/mining, beach nourishment, etc. constantly reshape the nearshore seafloor and influence the habitat substrate, of varying magnitudes over various time scales. The consequent alterations in benthic communities are frequently reported on changes to abundance, density, productivity, and biodiversity, which usually stretch further afield beyond the site of impact and may take indefinite time for recovery to pre-disturbance status^10^. The physical effects of regular tides or waves could be beneficial to particulate matter suspension and organic enrichment, under which circumstance filter feeders are adapted to thrive^11^. Nevertheless, severe storms generate near-bed currents and oscillatory flows that disrupt and move the substratum in which the benthos reside^12^. Exacerbated erosion, transport, and deposition processes of the sediment could directly cause substantial mortality or dispersal of infauna in the intertidal and the surf zone^13–15^. Negative biomass response to storms was observed most prominently for bivalves and crustaceans^16^. Figure 1 shows media reported photos of shellfish stranded on the shore after recent storm events. Yet this ecological response to the storm disturbance is incompletely understood.

Coastal clams are popular targets for recreational and commercial harvest^17,18^. Besides being an important source of food, clams play a vital role connecting microfauna and macrofauna in the benthic ecosystem^19,20^. Meanwhile, marine bivalves are highly vulnerable to multiple environmental stressors such as acidification, temperature changes, low-salinity exposure, pollution, and diseases^21–25^. More adverse effects and mortality are expected in the future due to the increasing human activities under the ongoing global climate change. Extensive research has been focused on the physiological effects of and adaptation to these biochemical stressors. However, the damage mechanism and vulnerability assessment of benthic bivalves subject to the physical disturbance process remain insufficiently resolved. Storm waves and surge can result in abrupt sediment instability of the seabed, which strongly affects the survival of bivalves and is directly related to the strength and duration of the event. Either excessive erosion or rapid accretion of the sediment was proved capable of causing great loss of buried creatures^26,27^. For instance, anchored clams, especially surface-dwelling juveniles, may be displaced, suspended, and passively transported along the bottom as bedload over relatively long time and distance, which is in proportion to the sediment flux, shear velocity, and turbulent kinetic energy^28,29^. This passive hydrodynamic dispersal process makes them more vulnerable to lesions and relocation failure^30,31^. On the other hand, clams would suffer significant smothering mortality due to the sediment overburden that exceeds their adaption limits^26^. The survival rate depends on factors such as burrowing ability, burial depth, sediment properties, and tolerance of anoxia^32,33^. The response of clams to sudden burial is vertically migrating upward until where the siphon may reach out the sediment layer. Therefore, both physical and biological knowledge are required to evaluate this kind of ecological vulnerability to the storm.

It is challenging to study the ecological effects of storms due to little data immediately before the event or limited post-storm sampling. An alternative avenue is to make reasoned prediction based the biological features of the species and their probable response to physical impacts on the habitat. The existing index methods based on the disaster exposure, physiological sensitivity, and recovery ability could provide tentative and qualitative vulnerability assessment, featuring rough hypotheses and assumptions^24,34^. In present work, numerical modelling of coastal hydrodynamics and sediment transport incorporating loss probability theory was applied to investigate the vulnerability of the clam habitat subject to storm waves and surge, through a case study at China’s northeast coast. The methodology is competent to map the storm damage distribution of the clam habitat across a landscape avoiding the labor-intensive field sampling. The study results are helpful to better understand the clam’s vulnerability to the storm disturbance and provide a useful approach applicable to the storm risk assessment of infauna communities in broader scopes.

## Study area and methodology

**Figure 2:**
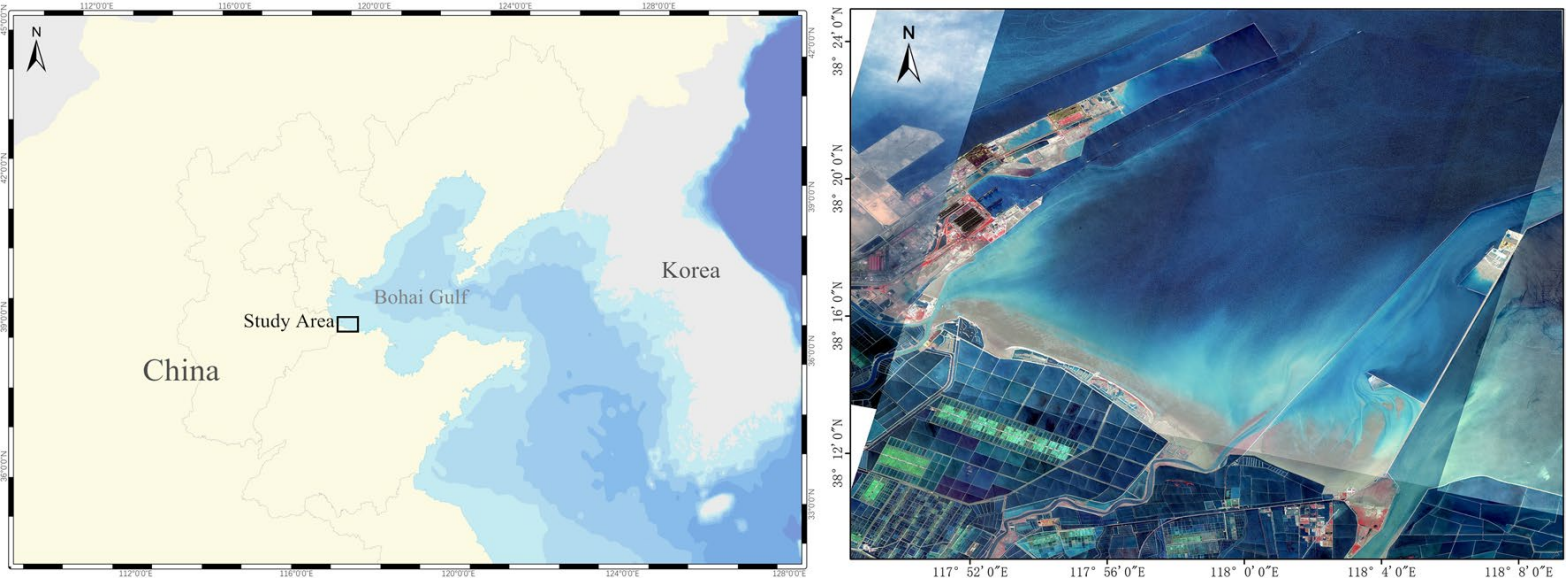
Location and GF2 satellite close-up image of the study area, August 2019 (processed by *ArcGIS 10.2* at https://www.arcgis.com/).

The study site, Binzhou Clam Habitat, is located inside the Bohai Gulf at China’s northeast coast as shown in Fig. 2. The mudflat with water depths below 5 m extends beyond 10 km offshore laterally bounded by two piers with two river outlets on the shore, adjacent to a coastal region largely covered by aquaculture ponds without industrial pollution. It is an ideal area for the reproduction and growth of clams with sufficient terrestrial nutrients input, to certain extent shielded from strong alongshore currents and waves. The habitat features an irregular semidiurnal tide with 1.82 m tidal range and 0.22 m/s tidal current’s velocity on average. The average local significant wave height is observed around 0.5 m with ENE and NE as main incident wave directions. The winter waves and extratropical storms are the most disastrous marine events including recent “Rumbia” in 2018 and “Lekima” in 2019. According to the granulometric analysis of samples, local seabed sediment size ranges from 4.9 to 60.9 µm with a median diameter (D50) of 26 µm, composed of fine sand, silt, and clay. The seabed surface layer contains abundant clams which was evidenced highly vulnerable to storm events. This study will establish a probabilistic correlation between the mortality/dispersal of buried clams and the depth of sediment erosion or deposition. Hydrodynamic modelling will then be used to simulate the sediment transport and net changes of the seafloor during the combined process of storm waves and surge. Eventually, the spatial distribution of the damage states will be evaluated according to the loss probability classification. The individual bedload movement of the clam is not separately addressed in the simulation, as it is largely represented by the macroscopic sediment transport.

## Loss probability analysis

Intensive substrate erosion by the hydrodynamics would lead to passive resuspension and transport of the anchored clams, which significantly decreases the survival chance by disrupting their comfortable status^27^. The turbid flow near the water–bed interface, directly related to substrate erosion-levels, may further hinder their respiration and intake processes. Although water flow in the immediate vicinity of clams stimulates the vertical mitigation behavior for better survival chance^35^. The dislodged clams could be unable to burrow back into the bed when the flow speed exceeds certain limits, usually ranging from 0.15 to 0.45 m/s corresponding to over 1.5 cm/s bed-shear velocity or TKE of 10.1 J/m^3^ for varying shell lengths^31,36–38^. On the contrary, abrupt excessive sediment deposition would increase the suffocation risk of the clams in greater buried depth before they were able to move into the upper layer.

Siphoning performance is vital for the living strategy of the buried clams. They breathe and feed by protruding the siphon to absorb oxygen and food from the overlying water. The burrowing depth mainly depends on the maximum stretching length of the siphon, which varies by species, age, temperature, and sediment type^39^. It might be analogical but arguable that burial depth is proportional to the bivalve size for the same species and shows significant seasonal variations, up to twice as deep in winter as in summer^40,41^. Interestingly in some research, medium clams show greater resistance to the erosion and more successful relocation compared to large ones, due to the fast reburial ability endorsed by higher siphon/shell weight ratio^29,42,43^. Such effect indicates a size threshold beyond which the clams are less adapted to the storm disturbance as the mobility decreases.

According to previous investigations, the clam burrows downward when the burying depth was eroded to less than 1.5 times the shell length, while moving upward when overburden with 2.5 times the shell length. The comfortable burying depth might be roughly estimated as 1.7–2.1 times the shell length^44,45^. Based on these biological propensity, we directly link the clam’s mortality to the sediment layer changes as the indicator of benthic ecosystem stability and hydrodynamic strength during a storm. A loss probability function, Eq. (1), is here proposed using multiple regression based on experimental data of existing works^40,46,47^. Major predictors are net seabed change ($\Delta h$) and clam shell length ($L$) while $P$ is the probability of death or dispersal. The shell length through different growth stage is described by Eq. (2) fitted to the sampled data in the study area, where $\mathrm{y}$ stands for the age. Parameters $\alpha$, $\beta$, $\gamma$ are representative for the clams in present study area and can as well be tuned for other bivalve species and sediment types elsewhere.

1 $$P=1-\mathit{exp}\left[-\gamma {\left(\frac{\Delta h}{L}\right)}^{2}\right]$$

2 $$L=\alpha {\mathit{tanh}\left(\frac{y}{5}\right)}^{\beta }$$

$$\alpha =5.3, \beta =0.64, \gamma =\left\{\begin{array}{c}0.172 erosion\\ 0.386 deposition\end{array}\right.$$

**Figure 3:**
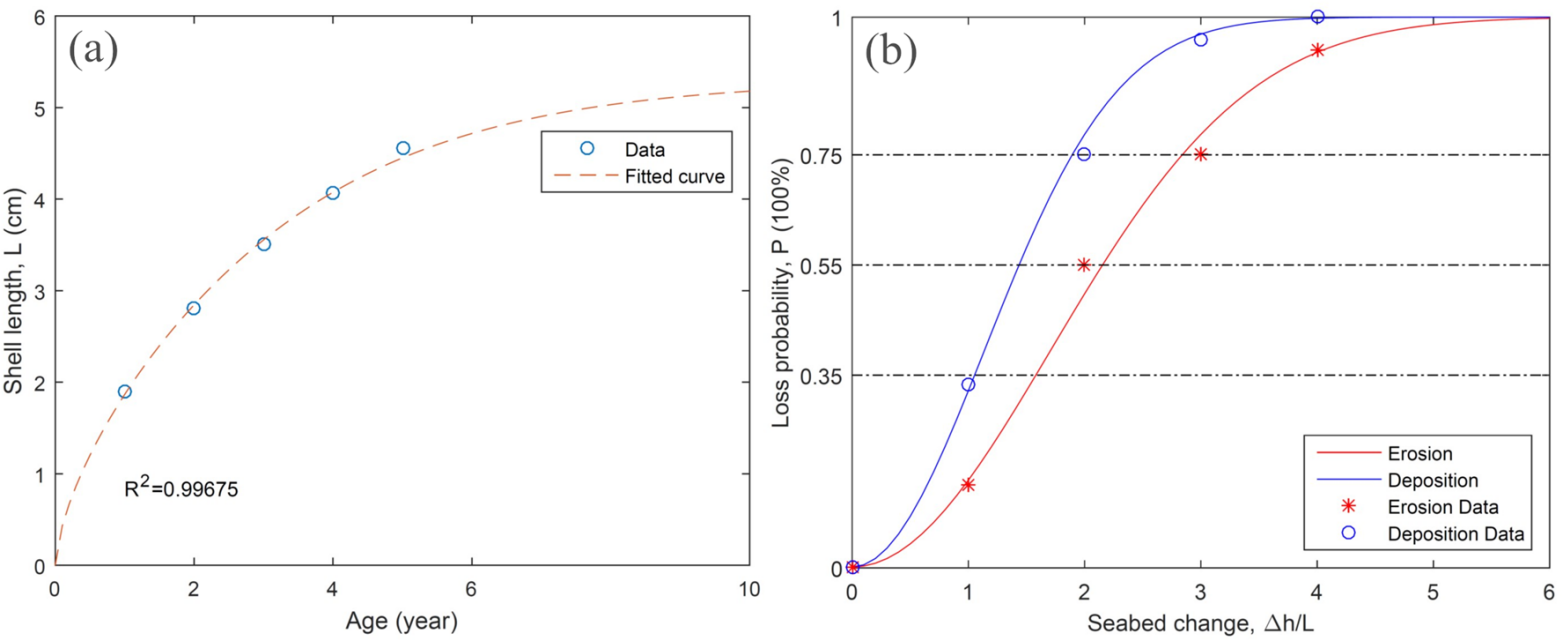
(**a**) Shell length of local clams at different growth phases evaluated by measured data and Eq. (2); (**b**) Expected loss probability based on experimental data and Eqs. (1–2) for abrupt change in sediment layer thickness.

As shown in Fig. 3a, the main harvest size of 2–3 years old clam in the habitat is about 3–4 cm. Figure 3b presents the loss probability curves of local clams under abrupt sediment erosion and deposition. Due to the strong vertical migration ability, the mortality only may reach 50% with an erosion depth of 2 times shell length that is about the maximum pre-burial depth. The loss rate rises to 78% and 90% as the erosion depth reaches 3 and 4 times shell length, in which case clams are very likely swept away by the near-bed flow. On the other hand, the sedimentation appears an easier death cause for pre-buried clams. An increased overburden layer of 1.5 time shell length would lead to a 58% fatality while 2 times shell length extra sedimentation features 78% loss probability. Four damage states are further classified by the loss probability criteria of 35%, 55%, and 75%, respectively. Once the seabed changes in a storm process are computed by the numerical model, the vulnerability assessment of the habitat would become much straightforward.

## Hydrodynamic modelling

### Configuration implementation

The WRF-MIKE21FM multi-scale coupling framework was applied to perform the storm event simulation^48^. The MIKE21FM consists of the NSWE-based Hydrodynamic module for currents and surge, the Spectral Wave module for wind-generated waves, and the Mud Transport module for cohesive/granular sediment movement^49^. The Weather Research and Forecasting (WRF) model provides the meteorological forcing at the air–water interface by hindcasting the wind, pressure and precipitation. The hydrodynamic model computes at the same time the circulation and the surge through the Saint–Venant equations. The storm surge is due to the pressure gradient and the wind stress, which are generally computed from the atmospheric forcing. Wave radiation stresses are computed by the spectral wave module and fed into the circulation module which is responsible for sediment transport and morphological changes. The near-bed shear velocity under a current is calculated using the standard logarithmic resistance law while the bottom oscillatory flow caused by the wave is approximated by the orbital velocity theory.

**Figure 4:**
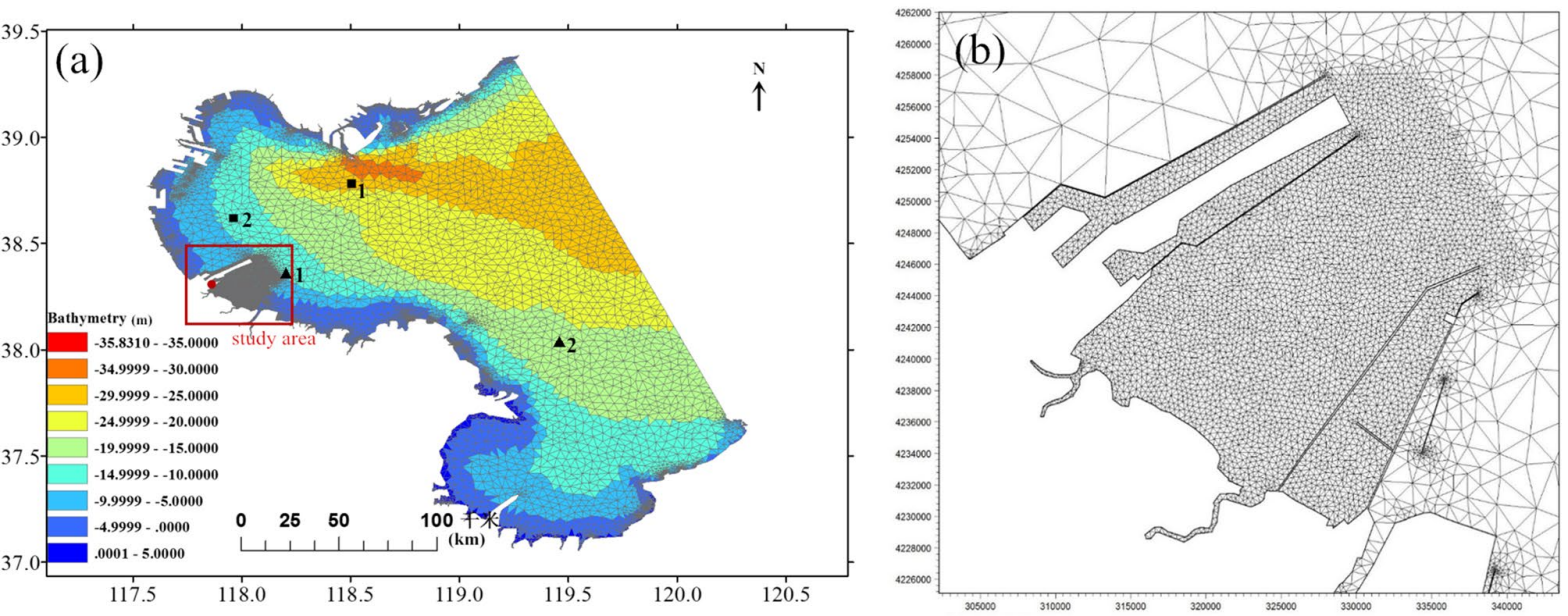
(**a**) Large scale unstructured mesh built on the bathymetry of inner Bohai Gulf, with locations of 2 wave buoys (black triangle), 2 current gauges (black square), and 1 tide station (red circle); (**b**) densified grids in the study area (processed by *Mike 21 at* https://www.mikepoweredbydhi.com/products/mike-21).

The initial and open boundary conditions of meteorology were taken from the Final Operational Global Analysis data of the U.S. NCEP. Data assimilation and three-level nesting were applied to construct the typhoon characteristics. The computing scheme of Galerkin finite element method was adopted with unstructured triangular grids over the bathymetry layer of the simulation domain (Fig. 4a). The mesh was locally refined at the study area with over 22,600 nodes and a 30 m minimum space step (Fig. 4b). The time step was set to be 0.3 s. The bathymetry data was interpolated based on the sea chart of CNHO (China Navy Hydrographic Office) and the engineering survey data of coastal projects, with respect to the mean sea level. Four major constituents of the astronomical tide (M2, S2, K1, O1) were used as the tidal forcing along the open-sea boundary based on the harmonic analysis. The characteristics of simulated storms (track coordinates, central pressure, wind speed, and radius of influence) were obtained from the track database of the National Meteorological Center. And the best hindcast results from the computation ensemble was selected. The theories and governing equations of these models are not iterated here while they can be found publicly in the official manuals and websites.

### Validation

**Figure 5:**
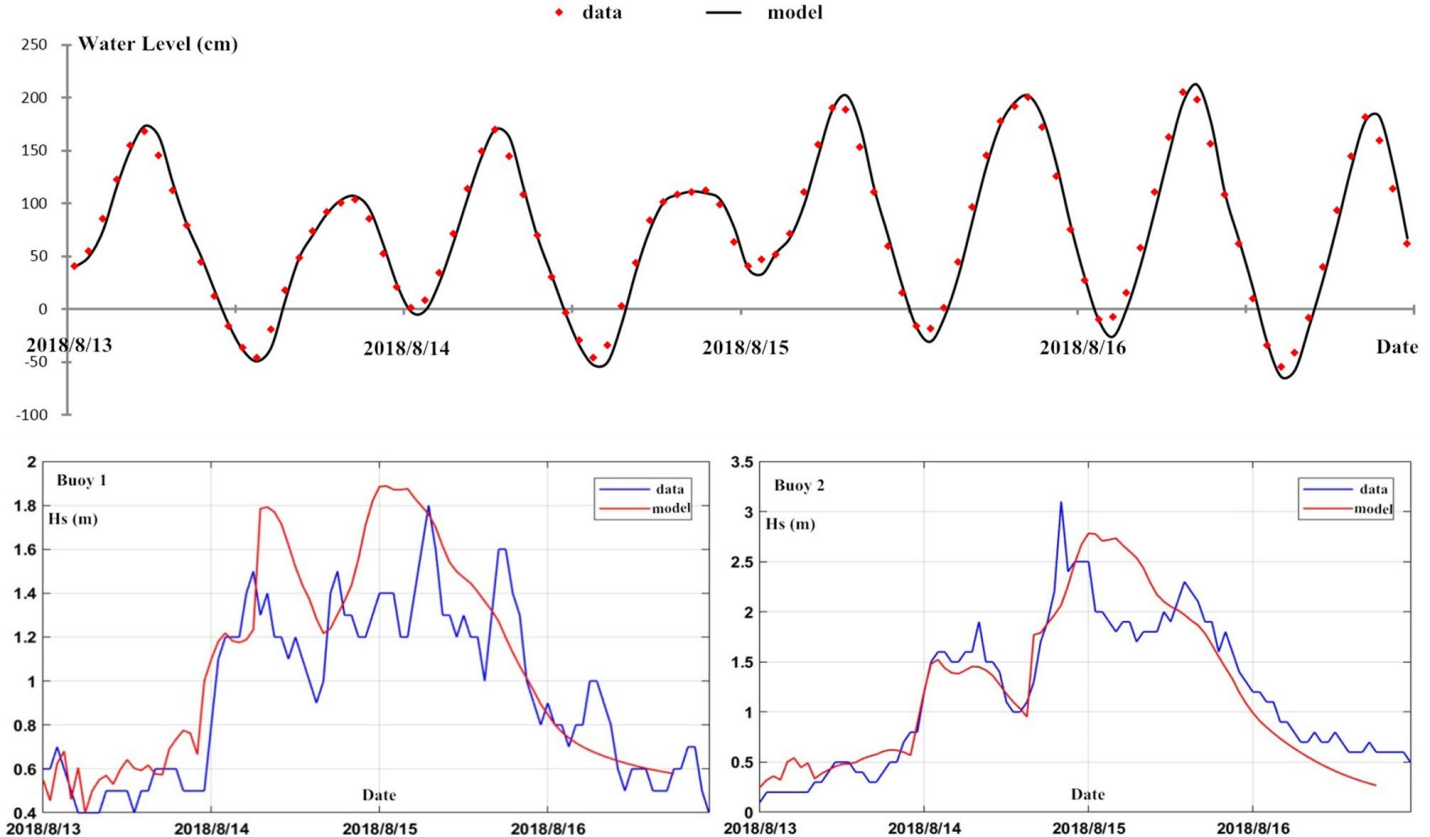
Time series of the computed storm tide (upper 2 panels) and waves (lower 2 panels) in comparison with observed data.

**Figure 6:**
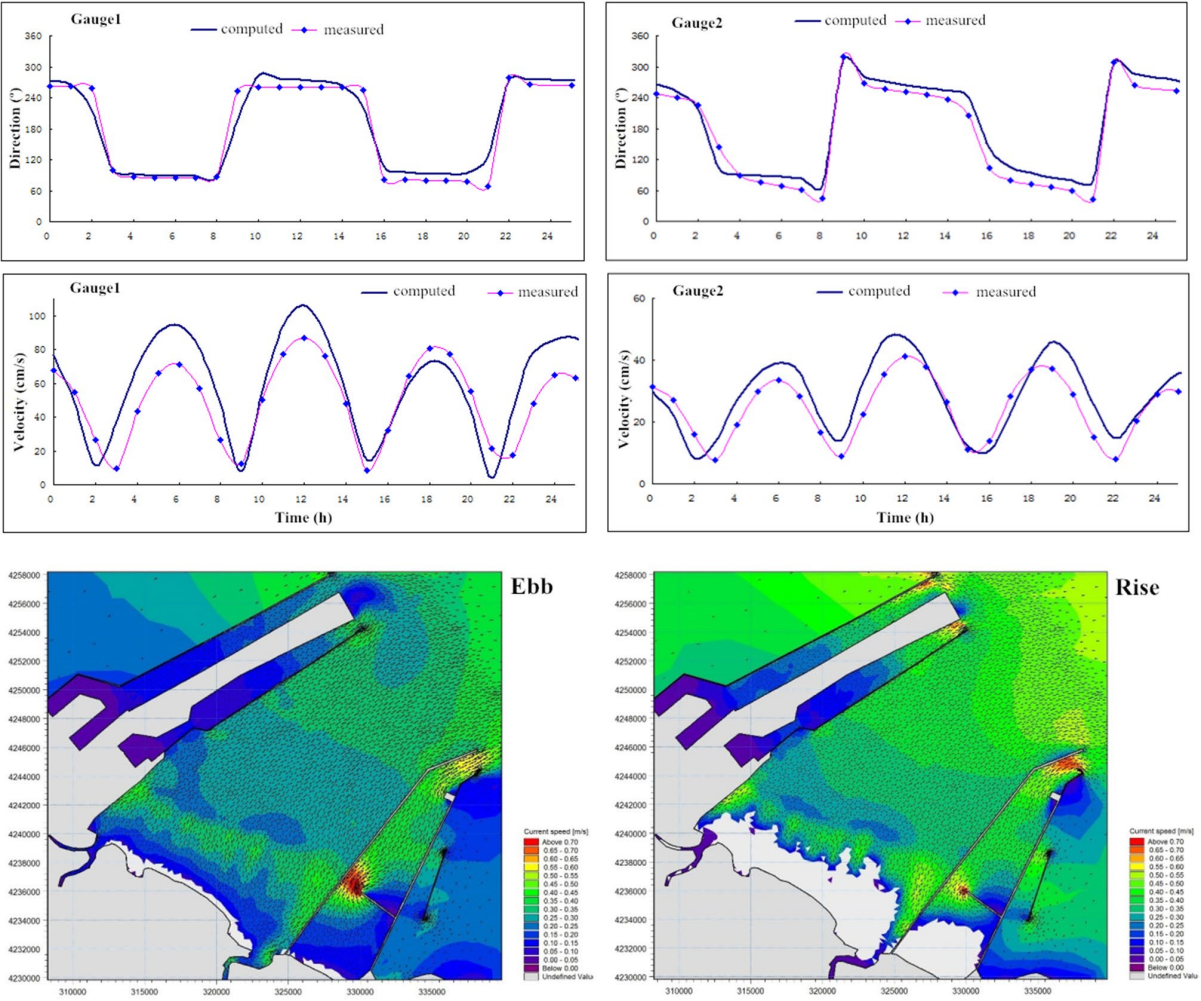
Time series of the current at 2 measurement points and the snapshots of velocity field at 2 tidal phases (processed by *Mike 21 at* https://www.mikepoweredbydhi.com/products/mike-21).

In order to ensure adequate computation of the hydrodynamics in the study area, the model was validated by comparing simulated storm surge, waves and currents to the observation data at gauge stations marked in Fig. 4a. Figure 5 demonstrates the computed time series of water level and wave height in comparison to observed data during the extratropical cyclones “Rumbia” and “Yagi” in 2018. The model shows excellent capability of capturing the amplitude and phase of the storm tide during two different time frames, with a good agreement with the observation at the tide station. The significant wave height as well presents quantitative consistency between the simulation and the buoy data with limited discrepancies. The upper four panels of Fig. 6 compare the simulated currents to the measurement data at the two gauge locations. The velocities and flow directions are computed well characterized with slightly overestimation in magnitude. Two snapshots of the velocity field for the study area during ebb and rise periods of the tide are respectively shown in the lower two panels of Fig. 6, featuring up to 0.5 m/s flow speed as the consequence of the mild topography. The bottom friction follows the Manning law with the spatial changing roughness coefficient n from 0.03 to 0.045 obtained by the calibration with tidal elevations. Validation results indicate the numerical model is fairly competent to predict the hydrodynamic environment during a storm event. However, sediment models feature more approximation/assumptions compared to the hydrodynamic theory and are highly sensitive to the computational accuracy of near-bed flows in coastal water. Therefore, sediment transport modelling is currently of limited accuracy, especially for short time duration and complex distribution of sediment composition.

## Vulnerability assessment

### Typhoon Rumbia

**Figure 7:**
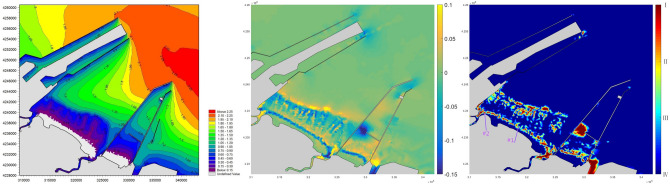
Typhoon Rumbia: simulated significant wave height; Computed net seabed changes by erosion and deposition; Damage state (I: highest, IV: lowest) assessment of clams with averaged shell length of 3.5 cm (processed by *Mike 21 at* https://www.mikepoweredbydhi.com/products/mike-21).

Typhoon Rumbia made its landfall at Shanghai on August 17, 2018 and entered the Bohai Sea from the mainland as an extratropical cyclone on August 20, 2018. The maximum storm surge was around 1 m at the study site. The left panel of Fig. 7 shows the significant wave height reached 1–2 m inside the habitat with two obvious wave shoaling/breaking belts 1 km and 5 km off the shoreline. The middle panel of Fig. 7 presents the computed spatial distribution of the net changes in the seabed right after the storm. Inside the habitat, the erosion depth ranges from 2 to 6 cm with a maximum of 8.3 cm, while the sedimentation thickness was mainly below 5 cm with a maximum of 7.2 cm. The right panel of Fig. 7 shows the damage states within the habitat domain assessed by incorporating the computed bed changes into the loss probability function, for the majority of the clams with averaged shell length of 3.5 cm. Level I (red) stands for over 75% loss while level IV (blue) represents less than 35% loss, in accordance with the criteria in Fig. 3b. The severe damage were scattered in the surf zone and mainly along the wave shoaling/breaking belts, where excessive erosion or overburden of the sediment was experienced.

To validate the accuracy of the vulnerability assessment, sampling verification at 2 distant locations, #1 (E117° 55′ 39.64", N38° 14′ 27.50") and #2 (E117° 51′ 36.82", N38° 16′ 11.75") in Fig. 7, were conducted 1 day before and after the storm. Four replicate core samples to the sediment depth of 15 cm were collected using a 25 cm quadrat at each location. The samples were sieved through a 2 mm mesh and the shell length of collected clams were measured around 3.5 cm in that season. Table 1 lists the clam counts at two locations before and after the storm. The loss rate are all above 75% at both sampling spots where the level 1 damage was predicted. The gradient of computed seabed change was not always smooth and with high-value points scattered. Those small vicinity area with singular large value would be covered by one color representing the highest level locally. The damage was more related to the erosion/dispersal at these two verification sites either from the computed results or the field sampling.

**Table 1:** Field sampling before and after the storm.

Location #	Quantity (before)	Quantity (after)	Loss rate (%)
1	66 in total	3 in total	95
	264/m^2^	12/m^2^	
2	86 in total	9 in total	89
	312/ m^2^	36/m^2^	

### Super Typhoon Lekima

**Figure 8:**
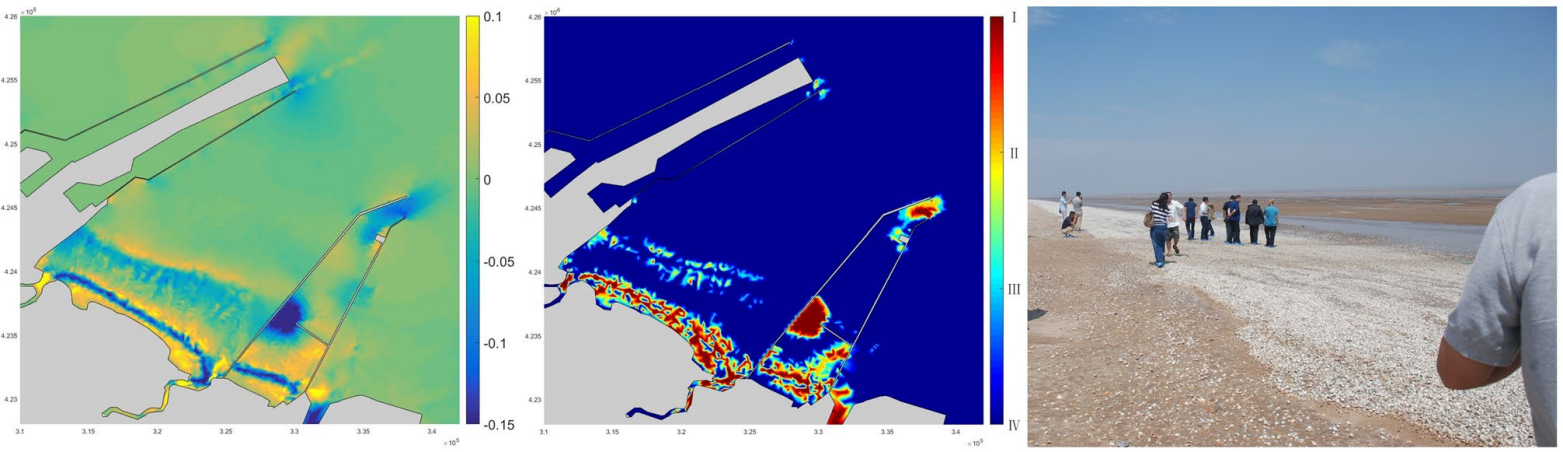
Storm Lekima: computed net seabed changes; damage state (I: highest, IV: lowest) assessment of the inhabiting clam with averaged size of 3.5 cm; post-storm “shell belt” of clams at the coastline (processed by *Mike 21 at* https://www.mikepoweredbydhi.com/products/mike-21).

Super Typhoon Lekima was one of the most destructive landing storms in history causing nearly 1.5 billion US dollar loss through 8 provinces and municipalities. The cyclone features maximum wind speed of 52 m/s at its first landfall on August 10, 2019 and swept northward across the mainland before eventually entering the Bohai Gulf on August 12, 2019. The storm surge was observed over 1.5 m with a 1.7–2.5 m significant wave height in the study area. Massive loss of the benthic creatures were reported at present clam habitat. The left plot of Fig. 8 shows the net bed changes are much more severe but with different spatial distribution compared to the Rumbia case. The erosion and sedimentation zones were laterally extended and parallel to the shoreline. The erosion depth ranges from 2 to 8 cm with a maximum of 13 cm, while the deposition thickness was mainly less than 6 cm with a maximum of 8.6 cm. The vulnerability assessment in the middle plot shows most of the loss was concentrated in the immediate vicinity of the shoreline, due to the high surge level that enables large waves to propagate very close to the coast before break. There was another damage belt a bit further offshore where the wave shoaling effect became strong. The right panel of Fig. 8 was a photo of the field survey taken 1 day after the storm. Tons of clams were strewn along the shoreline forming a “shell belt” which was consistent with the vulnerability prediction of the highest damage level.

## Discussion and conclusion

The lesion mechanism of benthic clam is complex and susceptible to many environmental factors such as temperature, salinity, pH value, pollution, and engineering activities. This paper studied the vulnerability of the clam subjected to the physical disturbance of extreme storm events. Abrupt excessive sedimentation and erosion of the substratum were identified as major causes of negative biomass response. This instability of the seabed results in either relocation failure after passive transport or suffocation in the anoxic overburden sediment for the buried clams. Based on the biological propensity and physiological sensitivity of the clam, we proposed a tunable loss probability function correlating the mortality with the shell length and the burial layer change in response to the hydrodynamic impact. Four damage states were classified by the specified loss probability criteria. The method could be further adapted to other bivalve species with different burrowing ability and burial depth. Because the net seabed change is an unobservable index, hydrodynamic model has to be applied to compute the sediment transport during the storm.

A clam habitat at the northeast coast of China was chosen for the demonstration study. The wind-surge–wave-sediment coupling model was adopted to simulate the hydrodynamic and morphodynamic processes for two recent high-impact storm events, “Rumbia” and “Lekima”. The model showed good performance in the validation of local tide, waves and currents. The magnitude of the erosion was computed generally less than the deposition in both storms. The estimated damage was mainly concentrated along the belt regions of wave shoaling and breaking, parallel to the shoreline within 5 km offshore. High surge levels pushed the damage belt shoreward as large waves were able to propagate very close to the shoreline before breaking. The vulnerability assessment results were preliminarily proved reasonable by the post-storm field survey. It is worth noting that the result of sediment transport is sensitive to the computational accuracy of near-bed currents and oscillatory flows in coastal water. However, the depth-averaged shallow water equations and the phase-averaged spectral wave model may underestimate the results of the highly nonlinear and dispersive wave-current dynamics in surf zone. Phase-resolving or nonhydrostatic models with good representation of depth-varying velocities could be considered for nested simulations in small nearshore domain. The field validation was the harsh part in this study. It was challenging to conduct intrusive and labor-intensive field sampling immediately before and after a storm with limited low-tide window period. The only 2 validation sites unfortunately featured the same damage level. More widespread sampling validation should be further conducted to improve the methodology by researchers who work on coastal ecosystem or mariculture that will withstand future marine disasters. Nevertheless, present work provides fundamental advance in understanding the clam’s vulnerability to storm disturbance and a utilitarian approach of the storm risk assessment for benthic communities in broader ecological and geophysical scopes.
